# Retaining infants and young children who experience transitions in care in longitudinal studies of child health and development: Considerations from the HEALthy Brain and Child Development study

**DOI:** 10.1002/imhj.70057

**Published:** 2025-11-26

**Authors:** Julie Poehlmann, Elizabeth I. Johnson, Pilar N. Ossorio, Keisher Highsmith, Brenda Jones Harden, Mishka Terplan, Pilar M. Sanjuan, Lorraine McKelvey, Claire D. Coles, Barbara H. Chaiyachati, Hon. Peggy Walker, Rebecca Shlafer, Kaitlyn Pritzl, Chandni Anandha Krishnan, Stephanie Averill, Samir Das, Santiago Torres‐Gomez, Florence Hilliard, Brian Gannon, Wesley K. Thompson

**Affiliations:** ^1^ Human Development and Family Studies University of Wisconsin Law School Madison Wisconsin USA; ^2^ Department of Family Medicine and Community Health University of Wisconsin‐Madison Madison Wisconsin USA; ^3^ Counseling, Human Development, and Family Science University of Tennessee Knoxville USA; ^4^ Division of Epidemiology, Service and Prevention Research National Institute of Drug Abuse USA; ^5^ Department of Human Development and Quantitative Methodology University of Maryland College Park USA; ^6^ Friends Research Institute Baltimore USA; ^7^ Family and Community Medicine University of New Mexico Health Sciences Center Albuquerque USA; ^8^ Department of Family and Preventative Medicine University of Arkansas for Medical Sciences USA; ^9^ Department of Psychiatry and Behavioral Sciences Emory University School of Medicine Atlanta USA; ^10^ Division of General Pediatrics Children's Hospital of Philadelphia Philadelphia USA; ^11^ Safe Babies Infant and Toddler Courts Atlanta Georgia USA; ^12^ Department of Pediatrics University of Minnesota‐Twin Cities USA; ^13^ Center for Human Development University of California San Diego USA; ^14^ Centre for Integrative Neuroscience Montreal Neurological Institute, McGill University Montreal Canada; ^15^ Department of Pediatrics The University of Alabama Tuscaloosa USA; ^16^ Laureate Institute for Brain Research Tulsa Oklahoma USA

**Keywords:** foster care, kinship care, non‐parental care, substance use, transitions in care

## Abstract

A transition in care (TIC) is a significant change in the primary adults who provide care for a child, involving a move to informal or formal non‐parental care, including kinship and foster care. In this paper, we address three issues: (1) the theoretical and empirical reasons for retaining infants and children who experience TIC in longitudinal studies of child health and development; (2) the import of retaining infants and children who experience TIC in studies focusing on parental substance use; and (3) methodological strategies for following children with TIC. We discuss the HEALthy Brain and Child Development (HBCD) study as an example of how a large prospective longitudinal cohort study can retain children who experience TIC, describing strategies such as: (1) documenting the frequency and contexts of these transitions and their associations with child health, mental health, and neurodevelopment; (2) attending to consent and mandated reporting requirements; (3) being sensitive to state child welfare policies and practices; (4) addressing retention challenges; (5) focusing on issues related to diversity, equity, and inclusion; and (6) establishing methods that document transitions and flexibly follow children as they grow older.

## 1 INTRODUCTION

Retaining infants and young children who experience transitions in care (TIC) in longitudinal studies is essential for understanding infant and early childhood development and mental health. TIC is defined here as significant change in the primary adults who care for children, including arrangements that are formal or informal, temporary or permanent, or occur within or outside of the foster care system. Previous studies have referred to TIC by different terms, including “foster care,” “out‐of‐home‐placement,” “non‐parental care,” “kinship care,” “kin and non‐kin foster care,” “relative or non‐relative placement,” “disruption in care,” “grandparents raising grandchildren,” and “skipped generation households” (e.g., Washington et al., 2021). In this paper, we use “TIC” as an umbrella term because it: (a) refers to a variety of care arrangements; (b) acknowledges that a child's reunion with birth parents also represents a transition; and (c) recognizes that many alternate caregivers play primary caregiving roles in children's daily lives. The phrase “TIC” includes children who are in the custody of the child welfare system (i.e., foster care, including formal kinship care), in addition to children cared for by relatives or other adults without the birth parents present in the households (e.g., children in informal kinship care, living with tribal members) (Testa, 2017; Washington et al., 2021).

Using the HEALthy Brain and Child Development (HBCD) study as an example, this article addresses three questions: (1) What are the theoretical and empirical reasons for retaining infants and children who experience TIC in longitudinal studies of child health and development?; (2) Why is it especially important to retain infants and children who experience TIC in studies focusing on parental substance use?; and (3) what are the best practices for following children with TIC? To answer these questions, this article explores: (1) the epidemiology of TIC in the United States (U.S.); (2) theoretical, conceptual, and empirical reasons for retaining infants and children who experience TIC, especially those who have experienced parental substance use, in child development research; (3) HBCD researchers’ approaches in design and implementation for including children who experience TIC; and (4) lessons learned and recommendations for researchers who wish to retain infants and young children experiencing TIC in their research. Abbreviations used throughout this paper can be found in Table 1.

**Key Findings**

- Multiple theories highlight how early caregiving instability has the potential to adversely affect young children's brain and behavioral outcomes. Epidemiologic data point to higher rates of placements among young children, especially those who are exposed to caregiver substance use. Developmental evidence underscores that early caregiver instability is associated with adverse outcomes across developmental domains.
- The Healthy Brain and Child Development study has demonstrated the feasibility of prospectively studying infants who experience transitions in care (TIC), including documenting the frequency and contexts of these transitions and their associations with child health, mental health, and neurodevelopment. To retain infants and young children who experience TIC, researchers should attend to consenting and mandated reporting requirements; be sensitive to state child welfare policies and practices; address retention challenges and issues related to diversity, equity, and inclusion; and employ methods that document transitions and flexibly follow children as they grow older.
- The HBCD study is likely to provide unprecedented opportunities to capture children's early TIC and to identify factors that mitigate risk and promote resilience, as well as methodological insights and best practices that can be used to inform future research.

**Relevance to Infant and Early Childhood Mental Health**

Retaining infants and young children who experience transitions in care in longitudinal population‐based studies is important for science and society, for both theoretical and empirical reasons. The Healthy Brain and Child Development study provides an exemplar for documenting transitions in care, and examining their impact on young children's brain and behavioral development. This is relevant to the field of infant and early child mental health (IECMH) as such studies have the potential to provide evidence regarding the impact of caregiving instability on young children's brain and behavioral development over time, particularly regarding their mental health outcomes. Practitioners in the IECMH field can use such evidence to increase their understanding of the mental health functioning of young children exposed to caregiver instability, who often need and receive IECMH services.

**TABLE 1:** Frequently used acronyms as they appear in this paper.

Frequently used acronyms as they appear in this paper	
Acronym	Full phrase
US	United States
HBCD	HEALthy Brain and Child Development
MRI	Magnetic Resonance Imaging
TIC	Transition in Care
NSCNC	National Survey of Children in Non‐parental Care
CAPTA	Child Abuse Prevention and Treatment Act
CPS	Child Protective Services
NSCAW	National Survey of Child and Adolescent Well‐being
IRB	Institutional Review Board
CABs	Community Advisory Boards
SOP	Standard Operating Procedure
LORIS	Longitudinal Online Research and Imaging System

The HBCD study is the largest long‐term longitudinal study of early brain and child development in the U.S., funded by the National Institutes of Health (Volkow et al., 2020). A primary objective of HBCD is to investigate how the brain develops in early childhood and is affected by prenatal and postnatal risk and protective factors, including prenatal substance exposure. The study begins prenatally and plans to follow children through 10 years of age. Rather than a nationally representative study, the sampling frame is designed to represent the population of people who give birth to infants in the sites’ catchment areas, with oversampling based on features of interest (i.e., prenatal substance use). HBCD samples three sub‐cohorts: (a) a general population group, reflecting diverse 18–50‐year‐old pregnant people; (b) a group with substance use (alcohol, nicotine, cannabis, or opioids) above a predefined threshold during pregnancy; and (c) a group demographically similar to the second sub‐cohort (b) but without prenatal substance use.

**FIGURE 1:**
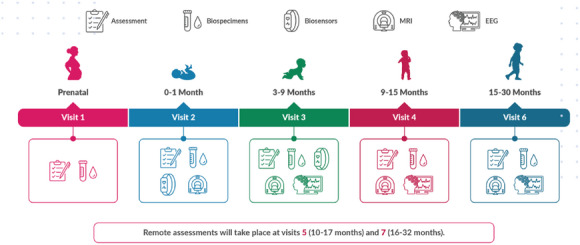
Timeline of HBCD study.

Research teams at 27 U.S. recruitment sites are enrolling about 7200 pregnant people and assessing them prenatally. Following birth, periodic assessments of the children and their caregivers (Figure 1) include structural and functional magnetic resonance imaging (fMRI); electroencephalogram recordings; anthropometric measurements; biospecimens; caregiver reports of children's developmental, medical, and family histories; direct assessments of child development; caregiver well‐being and home environments; and caregiver‐child interactions. Information derived from the HBCD study will be anonymized and made available to the broader research community via curated data releases starting in 2025. The HBCD study's design, especially the children's age range of birth to 10 years and the planned over‐enrollment of pregnant people who use substances, increases the likelihood that children will experience a formal or informal transition from their birth parents’ care during the study period. Given these factors, each HBCD site submitted a plan to follow children over time, even when they transition out of their birth parents’ care. Next, we briefly review existing research on the epidemiology of TIC, including its link to parental substance use.

### 1.1 Epidemiology of TIC in the United States

The evidence is clear: 21st‐century U.S. families reflect more diversity from demographic, cultural, and relationship perspectives (see Adler, 2023) than those from prior decades. Specifically, the 2020 U.S. Census found that 3 million children (4%) lived with neither biological nor adoptive parents (“non‐parental care”), growing from 2 million children (3%) in 1968, and increasing in racial and economic disparities (Hemez & Washington, 2021). In addition, a TIC for any reason (not just maltreatment) was more common for infants and very young children than for older children, with one‐third of U.S. children between 1 and 8 years of age living in non‐parental care (Radel et al., 2016).

Data from the National Survey of Children in Non‐parental Care (NSCNC), the only nationally representative survey of U.S. children living in all non‐parental care arrangements, indicate that most children in non‐parental care were living with grandparents (63%) or other relatives or family associates, such as aunts, godparents, or friends (22%) (Radel et al., 2016). Only 15% were living with foster parents, some of whom were related (i.e., foster care, including formal kinship care; Radel et al., 2016). Even though only a small proportion of these children are in foster care, they have been the most studied group of children experiencing TIC. Foster care placement typically occurs following a substantiated investigation of maltreatment (e.g., abuse or neglect) by the child welfare system. However, foster placement also occurs because of parental incarceration, child behavior or disability, parental substance use, or parental death (Drake et al., 2022). Racial and economic biases persist in the child welfare system, with more than half (53%) of African‐American children experiencing a child welfare investigation during their childhoods (Berger & Slack, 2020; Kim et al., 2017). Moreover, Black children are about twice as likely as White children to experience foster care placement (Wulczyn et al., 2023). American‐Indian/Alaska Native children are increasingly over‐represented in foster care (Roehrkasse, 2021).

Although the number of children coming into foster care nationally declined from 2018 to 2022 (Children's Bureau, 2023), there were geographic and age distinctions that affected rates of foster care placement over the last two decades (i.e., 2000–2018; Wulczyn, 2020). Specifically, there were increases in the foster care placement of children residing in non‐urban areas, including smaller metropolitan and rural areas. Relevant to this paper, children under the age of one year (especially newborns) experienced increases in the rate of foster care placement. Placement rates were particularly elevated for children who were below 1 year and living in non‐urban areas.

A family risk factor found to be associated with increased rates of children, especially infants, placed in foster care is caregiver substance use (Sepulveda & Williams, 2019). In fact, from 2007 to 2017, there was a 53% increase in the rate of children placed in foster care due to caregiver substance use (Williams & Sepulveda, 2019). This rise continued following the passage of the Comprehensive Addiction and Recovery Act (CARA, 2016), which amended the Child Abuse Prevention and Treatment Act (CAPTA) by requiring states to develop plans of safe care for “substance‐affected” children and to report information to the federal government on infants with prenatal substance exposures. CAPTA also requires states to mandate that health care providers involved in the delivery or care of children born with prenatal substance exposure notify child protective services (CPS) (CAPTA, 2024). In addition, states must have policies and procedures for referring (less formally termed “reporting”) such children for possible child abuse or neglect, when “appropriate” (CAPTA, 2024). A total of 45,756 infants in 50 states were referred to child welfare agencies in 2022 for prenatal substance exposure (U.S. DHHS, 2024a). Implementation of CAPTA notification and reporting laws varies across the states and the District of Columbia (Bruzelius et al., 2024; Morgridge Institute for Research, 2021). Families may also be referred to CPS for caregiver substance use after the child is born. CPS reports related to caregiver non‐alcohol substance use increased from 2005 to 2018, while reports for alcohol use did not change (He et al., 2022). Parental substance use is particularly associated with CPS involvement and placement in foster care for very young children (Williams & Sepulveda, 2019), which is highly relevant to following infants in the HBCD study, given its sampling frame.

Other factors that are often correlated with perinatal substance use increase the chances that a child will experience a TIC, including placement in foster care. These factors include family financial stress and conflict, parental intimate partner violence, parental mental health problems, family social isolation, and parents’ experiences of childhood maltreatment (e.g., Mulder et al., 2018; Stith et al., 2009). Most (i.e., three‐quarters) families in CPS with substantiated cases involve neglect, which is strongly related to poverty (Berger & Slack, 2020; Drake & Jonson‐Reid, 2013; Putnam‐Hornstein et al., 2013; U.S. DHHS, 2024a).

In general, infants and young children are more likely to experience maltreatment and subsequent foster care placement compared to older children. Children under 3 years represent the highest proportion of maltreatment victims (27.3%), with children under 1 year experiencing the highest rate of maltreatment (22.2 per 1000 children); moreover, children under 3 years are twice as likely as older children to be placed in foster care (U.S. DHHS, 2024a, 2024b; Williams & Sepulveda, 2019), and they are more likely to be placed for caregiver substance use. In 2017, 60.4% of children placed in foster care because of caregiver substance use were less than five years old, with 50.7% of them being less than one year old (Williams & Sepulveda, 2019).

The youngest children in foster care are also at risk for placement instability, which involves multiple TIC. For example, in a multistate study of foster care, infants were the most likely age group to reenter care after being returned home (Wulczyn et al., 2020). Across all age groups in the U.S., almost one‐quarter (22.3%) of children whose families were investigated for maltreatment were placed in foster care at least once in the 18 months post‐investigation, with 27.4% having two or more placements (National Survey of Child and Adolescent Well‐being (NSCAW); Dolan et al., 2013). However, 85.6% of children under the age of two had at least one placement change, and more than half (51.0%) had two or more changes (Casanueva et al., 2014). As we outline below, these transitions in primary caregiving can have significant implications for child development.

### 1.2 Theoretical and conceptual reasons for retaining infants who experience TIC in large developmental studies

The significance of including and retaining children experiencing TIC in studies of child development has been demonstrated by research guided by theories of attachment, developmental social neuroscience, social determinants of health, and developmental resilience. These research areas have demonstrated how TIC can affect child health and development in ways that should not be overlooked.

#### 1.2.1 Attachment theory

Attachment theory grew out of observations of the detrimental consequences of early parent‐child separation, as well as research in ethology and developmental science about normative infant‐caregiver interactions (Bowlby, 1982). Most often, infants develop secure attachments to their parents and other caregivers when they learn to trust that the adult consistently and sensitively responds to them in ways that keep them safe, alleviate distress or discomfort, and foster their exploration of the environment (Ainsworth et al., 1978; Bosmans et al., 2020). In contrast, insecure and disorganized attachments may develop under less optimal conditions, such as when caregivers respond to the infant inconsistently or insensitively, when fear or trauma is present in the relationship, or when disruptions in care occur. Disorders of non‐attachment, while rare and serious, can develop when an infant or young child does not have the opportunity to interact with a consistent adult who cares for them (e.g., being raised in an institution or under conditions of severe deprivation) (Guyon‐Harris et al., 2021; Zeanah & Gleason, 2015). Research with infants and young children who experienced early adversity in addition to placement in foster care found elevated rates of disorganized attachment, as well as developmental and mental health concerns (e.g., Vasileva & Petermann, 2018). The timing of disruption in care and transition to stable care also matters and infants adopted prior to 1 year of age showed rates of secure attachment similar to children who had not been adopted; however, when children were adopted after 12 months of age, they were less likely to develop secure attachments than children who were not adopted (van den Dries et al., 2009). As such, retaining children with TIC in research will allow HBCD investigators to further examine how changes in primary caregiving environments impact children's attachment experiences.

#### 1.2.2 Developmental social neuroscience

Developmental social neuroscience is the study of how social and biological factors interact throughout development, especially in children's brain development (Haan & Gunnar, 2009). Among the most comprehensive investigations linking children's early TIC to neural development is a study initiated more than 20 years ago with infants and toddlers in Romania who, between 7 and 33 months of age, were randomized from institutional care (with conditions of extreme neglect and deprivation) to family‐based foster care (Nelson et al., 2023) versus continued institutional care. The intervention had generally positive effects, with children randomized to foster care having better neurological outcomes than those who continued in institutional care (Nelson et al., 2023). However, it is yet to be understood how less severe TICs might impact brain development, and how other factors, such as the prenatal environment, interact with TIC.

#### 1.2.3 Social determinants of health

Social determinants of health models (e.g., Kaiser Family Foundation, 2018; Healthy People, 2030) have provided a framework to better understand how the well‐being of children is impacted by the environments where they live, learn, work, and play. These social determinants of health include economic stability, access to education, health care, neighborhood safety, and community contexts, and can also include social factors associated with TIC. A TIC often reflects but can also change the social conditions in which a child lives. For example, transitioning from the care of a parent who is unhoused into the care of a relative living in a home in a neighborhood with community resources shifts the conditions in which the child is living, learning, and playing. These models (e.g., Hunter & Flores, 2021) highlight the importance of retaining children through TIC in research that examines racial and economic disparities associated with TIC, including foster care (Harden et al., 2024).

#### 1.2.4 Developmental resilience models

Developmental resilience models highlight the importance of individual, family, community, and policy factors that may mitigate risk and promote positive child outcomes. While early conceptualizations of resilience focused on children's coping and individual‐level characteristics, current definitions emphasize resilience as a complex developmental process that unfolds over time and contexts (Masten et al., 2021; Ungar, 2018). Reviews of research on resilience factors suggest that sensitive caregiving, social support, and self‐regulatory skills may underpin positive developmental trajectories in the face of adversity (Masten et al., 2021; McLaughlin et al., 2020). Resilience frameworks also include factors beyond children and families, such as resources in schools and communities. Resources are often inequitably distributed, however, and critical approaches to resilience acknowledge these inequities, recognize the hidden physiological toll resilience can take, and advocate for systems‐level solutions to problems that necessitate resilience (e.g., Anderson, 2019; Murry et al., 2023). Retaining children with TIC in large longitudinal studies will allow further examination of individual, family, and community resilience factors.

### 1.3 Empirical reasons for including infants who experience TIC

Most research on children who experience TIC has focused on child welfare involvement, especially children in foster care. Much of the research draws from the National Survey of Child and Adolescent Well‐being (NSCAW), a longitudinal survey, now in its third iteration in the U.S., that is nationally representative of children involved with CPS. It includes data from children, parents and other caregivers, caseworkers, and teachers. The study focuses on measures of child and family well‐being and children and families’ experiences within CPS and their communities, but it lacks data prior to CPS contact, a gap that could be filled by large prospective longitudinal research studies that retain children who experience TIC.

In several NSCAW analyses, children who were placed in foster care exhibited more behavior problems than children who remained with their parents (e.g., Casanueva et al., 2012). However, out‐of‐home placement had little effect on children's behavior problems or cognitive skills after adjusting for “selection” factors (Berger et al., 2009). Additional analyses have contrasted types of care provided to children within the NSCAW dataset. For example, Casanueva et al. (2023) compared children and caregivers from three groups: voluntary/informal kinship care (although child welfare was involved, parents retained custody while children lived with relatives), formal kinship care (the state or Tribe had custody and the child was in foster care with a relative), and non‐relative foster care (the state or Tribe had custody and the child was in foster care with a non‐relative). On the one hand, children in kinship care arrangements fared better on several measures compared to children in nonrelative foster care, including better neurodevelopmental screening scores during infancy and toddlerhood, higher daily living skills in elementary and middle school, and fewer behavior problems in childhood and adolescence. On the other hand, voluntary kinship care providers were more likely to experience poverty, receive fewer support services, and have fewer financial resources compared to non‐kin foster care and formal kinship care providers.

Another source of information about children who experience TIC comes from the NSCNC. In the NSCNC, 1298 non‐parental caregivers completed interviews in 2013 about children's living arrangements, well‐being, and use of services. The most common reason for children living in non‐parental care was parental substance use (33.6%). The next most common reasons for non‐parental care in the full sample were maltreatment or removal by CPS for other reasons (14.6%) and parental incarceration (14.4%) (Radel et al., 2016). Data from the NSCNC has shown that 85% of children living in non‐parental care did so informally, most often with kin (Radel et al., 2016). Although most children in NSCNC who were living in formal foster care had a prior open CPS case, most of the other children did not have prior CPS involvement (Radel et al., 2016). Based on NSCNC data, children in non‐parental care were more likely than their peers in the general population to have mental health problems (16.5% vs. 27.1%), including depression, anxiety, behavior problems, Attention‐Deficit/Hyperactivity Disorder, learning disabilities, developmental delays, and intellectual disabilities. They were also more likely to have physical health problems (13.0% vs. 26.6%) including asthma, problems with vision and hearing, and epilepsy; they were less likely to have caregivers report that they were in excellent or very good health (84.2% vs. 79.2%) and were more likely than their peers to struggle in school (Radel et al., 2016). However, the NSCNC study involved data collection for children originally identified in the National Survey of Children's Health and did not follow children long‐term, representing a gap that could be filled by large longitudinal developmental studies that retain children through TIC, as the HBCD study is doing.

Despite the groundbreaking nature of these population‐based studies, many knowledge gaps remain. Researchers have not adequately assessed the impacts of experiences that occurred prior to children's TIC (including prenatal exposures), timing and length of transitions, reasons for transitions, the quality of alternate care received, and links between non‐parental care and children's brain development. Given these gaps, one goal of the HBCD study is to prospectively study young children who experience TIC over time, including documenting the frequency and contexts of transitions and their associations with child neurodevelopment and mental health.

### 1.4 HBCD approaches in design and implementation regarding TIC

To formalize efforts to achieve the goal of documenting the experiences and outcomes of children faced with TIC, HBCD study leaders established a TIC workgroup whose approaches are grounded in the theoretical and empirical literatures described previously. The TIC workgroup addressed both conceptual and methodological factors that would allow for the ethical and effective longitudinal examination of this subgroup of children. In the following sections, we describe the approaches used in the design and implementation of the HBCD study to facilitate the engagement, enrollment, and retention of children who experience TIC and their caregivers.

#### 1.4.1 Racial, ethnic, economic, stigma‐related, and other social inequities

HBCD investigators recognized that TIC most often occur in families experiencing multiple forms of marginalization (e.g., poverty, racial discrimination, stigma related to substance use). As such, it was critical that HBCD develop a comprehensive plan for retaining children who experienced TIC, thus allowing study findings to better reflect U.S. children's diversity.

To enhance the retention of children with TIC, HBCD aimed to recognize all family structures. Measures were edited so that terms such as “mother,” “father,” and “parent” were adapted to apply to a variety of caregiving environments and to include non‐biased language and gender inclusiveness (e.g., use of the term “caregiver”) so as to not make the common assumption that the birthing parent would be the primary caregiver throughout the study period.

#### 1.4.2 Consent and permission

Retaining children in a longitudinal study through TIC required HBCD researchers to determine whether and from whom they must obtain permission to continue the child's study participation. Each pregnant person enrolled in HBCD consents to their own participation and grants permission to enroll the child following the child's birth. Because the single Institutional Review Board (sIRB) responsible for HBCD has determined the study is minimal risk, permission from one parent is sufficient to authorize the child's participation (Code of Federal Regulations, 2024). In this context, the caregiver must have legal authority to authorize the child's research participation.

Transfer of legal decision‐making about a child from the parent(s) to another person occurs when there is: (1) initiation of formal foster care, (2) legal guardianship granted by a court to a non‐parent, (3) termination of parental rights, (4) voluntarily relinquishment of parental rights (in preparation for adoption), or (5) adoption. Whenever legal decision‐making power to authorize a child's research participation changes, researchers must seek new permission from the new authorized entity.

Foster care is a legal arrangement defined by each region and by the policies and practices of relevant agencies. When children enter formal foster care, typically the local or state Commissioner of Social Services (or an analogous government official) gains legal custody of the child, including authority to provide permission for a child's medical care and research participation (Szilagyi et al., 2015). In most states, the Commissioner may delegate that authority to other child welfare personnel, though there are some notable exceptions. In some states, research involving children in foster care must be reviewed and approved by a separate state IRB (see, for example, Illinois Department of Children and Family Services, 2024). Foster parents generally do not hold decision‐making authority for children in their care.

When a termination of parental rights becomes final, or when parents have relinquished parental rights voluntarily, the child becomes a ward of the state (a legal orphan), with only the state having rights to the child until permanency occurs through adoption. Thus, the process of obtaining new permission for such children's continuing research participation is similar to that required for foster children. The U.S. DHHS regulations specifying additional protections for children in research, 45 C.F.R. 46 Subpart D, contain special protections for children who are state wards (U.S. DHHS, 2024). Researchers who seek to retain children through transitions into formal foster care must ensure that their study meets these regulatory criteria and that the IRB has approved the study's inclusion of foster children.

Guardianships for children can vary from emergency to temporary to long‐term (see Lauck & Roelandts, 2021). Guardianship may occur with or without consent of the legal parent(s), and a child who has a guardian is not considered a ward of the state. Guardians must be appointed by a court, and they obtain some or all legal rights and duties a parent usually has in relation to their child. Guardianship by a child's relatives has become increasingly common (Smith, 2019), as the federal Guardianship Assistance Program under Title IV‐E of the Social Security Act grants states and tribal organizations money to provide financial assistance for the care of children whose relatives have assumed guardianship (Administration on Children, Youth and Families, Children's Bureau 2024). If a child has an alternate caregiver, researchers should ascertain whether that person has been appointed by a court as the child's legal guardian, and if so, whether the guardianship includes authority for medical and research decisions on the child's behalf. If the answers to those questions are “yes,” then researchers must obtain the guardian's permission for the child to continue in the study, as the original parental permission is no longer operative.

However, an informal TIC does not automatically grant the alternate caregiver legal authority to make important decisions on the child's behalf. Researchers should not assume that a birthing parent who enters residential treatment for substance use, is incarcerated, or is otherwise not residing with the child has lost authority to make decisions about the child's participation in research. Of course, even temporary alternate caregivers have a practical veto over the child's research participation because they typically determine whether the child will attend research visits. Also, if the alternate caregiver is asked to answer questionnaires, then that person becomes a research participant and must consent for themselves.

**FIGURE 2:**
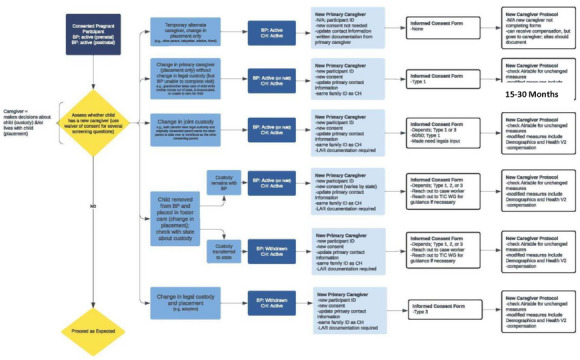
Flowchart used to determine alternate caregivers in the HBCD study.

To help HBCD researchers determine when new permission is required for a child's study participation, and from whom it is required, the TIC workgroup created a decision flowchart, with different types of consent/permissions for each caregiver type (Figure 2).

#### 1.4.3 Child welfare system involvement

Because child welfare involvement often overlaps with children's TIC (and pre‐ and post‐natal parental substance use), the National Institute on Drug Abuse (NIDA) provided funding for a workshop on the child welfare system prior to HBCD data collection https://heal.nih.gov/news/events/engaging‐child‐welfare‐systems. The workshop provided an overview of the child welfare system, including a discussion with parents who had lived experience of child welfare involvement. Subsequent sessions summarized current child welfare policies and practices including documented racial, ethnic, social and economic biases; empirical evidence on intersections among substance use, the criminal legal system, and the child welfare system; and research on the impacts of poverty, maltreatment, and foster care on children's brain and behavioral development (Detlaff, 2020; Putnam‐Hornstein et al., 2013). Workshop presenters offered recommendations for research implementation.

Building on workshop recommendations in addition to HBCD investigator expertise and input from sites’ Community Advisory Boards (CABs), the TIC workgroup developed a standard operating procedure (SOP) to help HBCD sites integrate knowledge about the child welfare system into their research practices (Appendix A). SOP recommendations fell into three categories: (1) strategies to engage participants with child welfare system involvement, (2) development, within research teams, of expertise concerning the child welfare system, including child maltreatment reporting, and (3) issues related to research questions and data collection methods.

The SOP recommends that all staff (i.e., PIs, program coordinators, research assistants, students) be well‐informed about the child welfare system and ethical considerations. Site research teams were encouraged to invite people who have lived experience of substance use and/or child welfare to serve on their CABs, as well as to establish connections with local child welfare agencies, including identifying liaisons to facilitate retention of children with TICs. The SOP also recommended establishing Memoranda of Understanding between investigators and the child welfare systems, which address how to handle maltreatment reports and follow children in foster care.

Knowledge of state statutes for mandated reporting of child abuse and neglect is crucial (Child Welfare Information Gateway, 2023). Laws regarding mandated reporting differ by state, with some states considering all people as mandated reporters and other states limiting this requirement to certain professions (Morgridge Institute for Research, 2021). The HBCD study includes sites in 23 states, and despite the heterogeneity among maltreatment reporting laws, HBCD considers researchers at all study sites as mandated reporters of child abuse and neglect. However, HBCD defers to state statutes regarding definitions of child maltreatment, because such definitions vary across states, particularly for parental substance use (as we discuss below). The SOP also outlined procedures for the determination of possible child maltreatment, emphasizing three points: (1) research staff should pause (unless there is an emergency) before presuming parenting behavior is maltreatment; (2) researchers should support all participants in a sensitive and inclusive manner; and (3) staff need support around issues related to child maltreatment, reporting, and trauma. The pause mentioned in (1) permits study personnel to assess whether their information about a participant truly meets their state's legal criteria for mandated reporting. If not, then a federal statute pertaining to the privacy of research data would bar study personnel from making a report (US Code, 2018).

Children and families experiencing TIC may be stressed, and researchers should not add to such stress by treating them disrespectfully or exacerbating stigma. Thus, the SOP emphasizes the use of non‐stigmatizing language regarding families impacted by child welfare, legal system involvement, or substance use, and staff engagement in ongoing unconscious bias training.

#### 1.4.4 Prenatal substance exposure and mandated reporting

Because of HBCD's sampling frame, a critical ethical and study‐design concern involved states’ statutes mandating CPS notification or report of children born with prenatal substance exposure, as the report of a substance‐affected newborn to CPS is often the reason for newborns’ TIC. State statutes vary regarding who has such a mandate and what evidence triggers it. In every state, health care providers involved in newborn delivery or care must notify or report, but additional people may also face a mandate, including research team members. The extent to which notifications or reports occur reflects providers’ judgments and culture, health care institutions’ policies, and city‐ and county‐level enforcement decisions.

Nearly half of the states in the U.S. explicitly criminalize substance use during pregnancy (Bruzelius et al, 2024). Even when such use is not criminalized, the law can be “punitive” if it focuses primarily on removal of infants from parental custody or termination of parental rights rather than on treatment for substance use or referral to other services (Bruzelius et al, 2024). However, numerous professional societies have published statements opposing criminal and punitive policy approaches to parental substance use (Bruzelius et al., 2024).

In order to reduce the chances that participation in HBCD would lead to additional TIC, before collecting data, HBCD researchers proactively addressed participants’ legal risks related to prenatal substance exposure and designed the study to minimize the possibility that personnel would be required to report pregnant participants or their substance‐exposed infants to CPS. The study does not collect types of data (e.g., newborn toxicology) that trigger a mandated notification or report in many states. Further, using technological and procedural means at study sites where prenatal substance use is mandated to be reported, information that could trigger a mandated report is not accessible to research team members.

HBCD investigators and other researchers will eventually have access to large datasets containing all or nearly all data collected during the study, including measures of substance use during pregnancy. However, HBCD researchers will not have the ability to readily link individual‐level data in a released dataset to site‐level identifying information. In addition, researchers and their study personnel who are provided access to HBCD data will sign a data use certificate in which they agree, among other things, not to attempt to identify anyone in the dataset. By investing years in understanding the legal risks and designing the study to avoid them, the HBCD study has decreased, to near zero, the likelihood that participation in the study will trigger a TIC solely due to prenatal substance exposure.

#### 1.4.5 Recruitment and retention

For pregnant people who use substances, barriers in seeking prenatal and postnatal healthcare also create challenges for participant recruitment. Stigma, shame, and negative experiences from healthcare providers and others have historically created barriers of mistrust for those who have a substance use disorder during pregnancy (Room, 2005; Stone, 2015; Hilliard et al., 2023), and punitive laws may keep parents from seeking help. Overcoming mistrust with populations that are substance involved requires innovative models that build trust and create additional ways to engage and retain participants.

To answer this challenge, the HBCD study developed Study Navigator positions to enhance recruitment and retention, modeled after the Substance Abuse and Mental Health Association guidelines for Certified Peer Support Specialists. This is a paraprofessional qualification for those who have lived experience with substance use disorder, are in recovery, and are trained to work in settings that help participants navigate study involvement. In the HBCD study, the Study Navigator is an integral part of the research team and works to build trusting relationships with participants, connecting them with resources. A recent randomized study, conducted during the study's planning phase, showed that involving peer support specialists as research team members increased recruitment and retention among participants with a history of prenatal substance use disorder (Zgierska et al., 2023). The HBCD Study Navigator model has now grown to include staff with other related human service qualifications. The Study Navigator can also help build trust between the study team and the families, which can be helpful if a TIC occurs. As members of the study team, Study Navigators are all trained in the ethical engagement of human subjects in research and are mandated reporters. Study Navigators follow SOPs and work in teams, thus reducing individual discretion about whether concerns about children's safety require a report to CPS. In addition to the Study Navigator model, HBCD has recommended that sites partner with community organizations that have already built trusting relationships with pregnant and postpartum people experiencing marginalization. Following study enrollment, such community organizations may be helpful regarding retention. For families involved with the child welfare systems, these community agencies may deliver interventions and provide support to families to prevent maltreatment and subsequent placement in foster or kinship care (Harden et al., 2020).

Multiple logistical challenges exist when attempting to retain children who experience TIC in research. A host of HBCD study retention strategies have focused on creating a warm, familiar, and trusting environment for participants, with emphasis on building positive relationships with families (Harden et al., 2024). Study staff stay connected through various “touchpoints” (e.g., checking in at certain times via phone calls, social media, or sending birthday cards). HBCD study researchers collect alternate contacts for participants at enrollment, such as contact information for family members or friends of the birth parent. The alternate contacts can be contacted if the research team is unable to communicate with the birth parent, which may happen when a TIC occurs. The IRB has issued a waiver of consent for these brief contacts, which help researchers locate a child. Researchers also gather social media information, which is less likely to change than addresses. At some sites, paid services are used to update address information through the U.S. postal service (which participants can opt into using a checkbox on the consent form).

#### 1.4.6 Methodological considerations

Regarding data organization, HBCD is designed with flexibility to follow children through different care arrangements over time. Many national population‐based studies that include child participants assign an identification number (ID) to the parent's research profile, and children are organized based on the parent's ID. In contrast, the HBCD study assigns IDs to children, while parents’ and alternate caregivers’ profiles are associated with child IDs. This flexible design feature allows researchers to follow children as they transition in and out of alternate care arrangements and to collect data from the adults who directly provide children's day‐to‐day care.

To do this, HBCD utilizes a centralized, multisite, multimodal, web‐based data capture platform, Longitudinal Online Research and Imaging System (LORIS; loris.ca; Das et al., 2012), to aggregate data and streamline workflows for structured dissemination. To enhance caregiver transition functionality, the HBCD study's LORIS team adapted the existing data capture platform by (1) repurposing the dual‐function Cohort field, and (2) working with the TIC workgroup to develop a new screening instrument. Specifically, the Cohort field was repurposed to serve a dual function, providing information on caregiver type during transitions. Previously, caregiver changes were not reflected in the assessment battery; now, customized batteries are implemented based on caregiver type, replacing the previous method of annotation. In addition, a new instrument (i.e., TIC Screener) was developed to facilitate this repurposing. An essential aspect of interoperability involves linking to Ripple, an external patient registry, which updates the LORIS instrument with relevant data. This process required the creation of a dedicated Application Programming Interface endpoint for full automation and the establishment of SOPs tailored to this workflow. The workflow is also applicable to complex scenarios involving multiple birth participants, ensuring comprehensive data capture and management. Using these systems, research staff can create a new caregiver profile associated with the child's profile once a TIC occurs. The original caregiver is placed on “hold” yet retains the ability to become an active study participant again if reunited with the child.

In terms of measurement, the workgroup developed several measures to gather data about children's TIC, including the TIC Screener discussed above. Research assistants administer the TIC Screener to birth parents or alternate caregivers at each timepoint after the child's birth, inquiring about whether or not a transition occurred, and if so, when the transition occurred and the reasons for the transition, with whom the child is currently living, and who has legal custody or guardianship of the child.

In addition, a TIC Interview, administered by research assistants at Visit 6 (Figure 1), when children are between 15 to 30 months, asks about any transitions that have occurred since the child's birth, including when the transition(s) occurred, the length of each transition, reasons for the transition(s), and the relationship of the caregiver(s) to the child (Table 2). The interview is designed to capture transitions that occurred between visits or that were not previously recorded. To further explore reasons that children may experience TIC, an Incarceration History Questionnaire is administered to children's caregivers at Visit 7, which occurs remotely when children are between 16 and 31 months.

**TABLE 2:** Definitions of alternate caregivers created in HBCD.

Caregiver type	Definition
Type A	Temporary alternative caregiver
Type B	Change in primary caregiver without change in legal custody (change in placement only, but birth parent unable to attend visit)
Type C	Change in joint custody
Type D	Child placed in foster care
Type E	Change in legal custody and placement (e.g., adoption)
Type F	Original consented parent

Alternate caregiver consent type	Definition
Type 1	Caregiver signs the consent form for their own participation only
Type 2	Caregiver signs the permission form for the child's participation only
Type 3	Caregiver signs consent form for their own participation and signs permission form for child's participation

Analyzing longitudinal datasets that include children who do and do not experience TIC presents both challenges and opportunities. A major challenge to handling caregiver transitions in longitudinal analyses lies in variation across caregivers who report on the child. Different caregivers have different perspectives, which may reflect varied responses regarding the child's environment or behavior. Longitudinal analyses of such data need to account for potential differences arising from changing caregiver reporters across time. Specifically, variation in caregiver answers regarding the child's behavior or environment may or may not reflect actual changes in the child's behavior or environment. In these circumstances, it may be necessary to encode aspects of the caregiving environment into characteristics or categories relevant for parsing real developmental variation across time versus changing caregiver perspectives.

To isolate the causal effects of TIC on subsequent child outcomes, one must rigorously address both confounding and reverse causality. One notable strength of HBCD is that data are collected prenatally, *before* any TIC occurs. This prospective, prenatal design of the HBCD study provides a unique opportunity to observe pre‐TIC characteristics, allowing for adjustment of baseline covariates that may confound associations between TIC and developmental outcomes, and to examine mediators and moderators over time. Analytical strategies such as marginal structural models or targeted maximum likelihood estimation can flexibly account for time‐varying confounding and selection bias. Further, leveraging within‐child longitudinal variation with fixed effects or random‐intercept cross‐lagged panel models can mitigate bias from unmeasured time‐invariant confounders. Causal identification requires careful temporal mapping of exposures, outcomes, and transitions, along with explicit modeling of the processes that predict TIC, potentially using inverse probability weighting or g‐methods to correct for endogenous selection into care transitions.

### 1.5 Summary, lessons learned, and recommendations

In summary, successfully documenting the experiences and outcomes of children who contend with TIC requires multiple strategies. We are employing the following strategies in the HBCD study: (1) documenting the frequency and contexts of these transitions and their associations with child health, mental health, and neurodevelopment; (2) attending to consent and mandated reporting requirements; (3) being sensitive to state child welfare policies and practices; (4) addressing retention challenges; (5) focusing on issues related to diversity, equity, and inclusion; and (6) establishing measurement, data organization, and data analytic methods that document transitions and flexibly follow children as they grow older.

However, this endeavor presents distinct challenges, particularly concerning CPS and the criminal legal system. Concerns persist regarding the efficacy of the child welfare and criminal legal systems in meeting the needs of families, as these systems occur within structures of surveillance, regulation, and punishment, typically without resources to address underlying issues (Williams, 2024). These issues are compounded when parental substance use is involved. Moreover, these systems are encumbered by racial biases, stigmatization of mental health disorders, trauma, and poverty, and bureaucratic hurdles that perpetuate racial/ethnic inequities. Communities with more frequent interactions with CPS and law enforcement may experience strained relationships and decreased trust, hindering rather than promoting compassionate care. The HBCD study recommends embracing the “mandated supporting” framework, which centers families through equitable, trauma‐informed practices. Our community engagement approach, driven largely by our Community Advisory Boards and Study Navigators, allows this to occur in the context of a complex, longitudinal study. The approach to the HBCD study as a whole, as well as the TIC protocols, underscores the intricate dynamics involved in engaging and retaining infants and their caregivers in research who experience adversity, particularly amidst TIC.

The lessons learned from the HBCD study highlight numerous research opportunities and challenges with engagement and retention of children who experience TIC. To address some of these challenges, HBCD has established a comprehensive strategy encompassing: (1) a values statement regarding inclusion of all children and families; (2) a broad definition of caregivers and appropriate screening and consenting procedures for different caregiver types; (3) training for study teams on the child welfare system, unconscious bias, and stigma; (4) involvement of individuals with lived experience and deep knowledge of the child welfare system; (5) use of a Study Navigator model to facilitate recruitment and retention of marginalized families; (6) alignment of an adaptable data organization platform, measures, and planned analyses to accommodate children's TIC; and (7) compliance with state and institutional research regulations for inclusion of children prenatally exposed to substances and their families (e.g., training, SOPs, study design) (see Table 3).

**TABLE 3:** HBCD's comprehensive strategy for retaining infants who experience transitions in care.

Strategy	Implementation
Values statement regarding inclusion of all children and families	Posted values statement on the HBCD website, which each study team member must read and sign Workgroups discussed recruitment and retention strategies to attain goals Workgroups and administrative core personnel monitor data dashboards weekly regarding demographics and adjust strategies accordingly Diversity, equity, and inclusion (DEI) Workgroup reviewed all measures and research protocols
A broad definition of caregivers and appropriate screening and consenting procedures for different caregiver types	Created a Transitions in Care Workgroup Developed workflows and data organization strategies that support the participation of a range of caregivers Used broad language in consents and measures to ensure inclusiveness and non‐stigmatization Developed screening measures that can detect transitions that occur at birth and throughout the infant's development Collaborated with trusted community organizations that serve diverse families, especially those experiencing marginalization
Training for study teams on the child welfare system, unconscious bias, and stigma	Initial and ongoing training required for staff regarding DEI Initial national training offered regarding child welfare, with access to the video to onboard new personnel Initial and ongoing training offered regarding site‐specific child welfare issues Developed a child welfare SOP, with special attention to the ethics and process of mandated reporting at each study site
Involvement of individuals with lived experience and deep knowledge of the child welfare system	Included people with lived experience of substance use and child welfare involvement on Community Advisory Boards (CABs) at each study site Included child welfare liaisons in CABs at each site
Use of a Study Navigator model to facilitate recruitment and retention of marginalized families	Support participants and their families in the context of prenatal substance use Use a “mandated supporters” framework (e.g., Mandated Supporting — JMACforFamilies) Adopted retention strategies that are trauma‐informed and comprehensive Initial and ongoing training of staff is required regarding the use of non‐stigmatizing language
Alignment of an adaptable data organization platform, measures, and planned analyses to accommodate children's changes in care	Developed and implemented flexible data organization systems that allow children to be followed as they transition to different caregivers Developed measures designed to capture details about children's transitions in care Considered the complexities of children's transitions in care and different caregiver types for planned analyses
Compliance with state and institutional research regulations for inclusion of children prenatally exposed to substances and their families	Reviewed laws in study site states in the planning phase and adjusted study procedures to ensure compliance with requirements Developed standard operating procedures and implemented site‐specific data sharing agreements regarding mandated reporting

Further, our approach to the HBCD study is grounded in the theoretical perspectives delineated previously in this paper. For example, we will be able to examine TIC and their relation to the attachment experiences of children, specifically through protocols that address stranger anxiety and the quality of parent‐child interactions. Clearly, the multiple assessments of brain functioning, which can be examined in the context of TIC, have implications for theory and research in developmental social neuroscience. Because the study is addressing myriad social determinants of health, including TIC, we will be able to document the additive and interactive influences of these determinants on children's physical and mental health. Finally, the HBCD study is squarely situated in developmental resilience models, as we will be able to document not only the risks that precede and follow TIC, but also the individual, family, community, and policy factors that mitigate risk and promote positive child outcomes.

Finally, our approach regarding children who are experiencing TIC has significant implications for infant and early childhood mental health practice and research. Most importantly, such studies have the potential to provide evidence regarding the impact of caregiving instability on young children's brain and behavioral development over time, particularly regarding their mental health outcomes. Practitioners in the IECMH field can use such evidence to increase their understanding of the mental health functioning of young children exposed to caregiver instability, who often need and receive IECMH services. The interactive experiences and effects of prenatal exposures (e.g., substances) and maternal mental health during pregnancy are critical considerations for perinatal mental health providers. Additionally, the role of the post‐natal caregiving environment on young children's brain and behavioral outcomes is central to an IECMH approach. Evidence from the HBCD study has the potential to inform how IECMH providers, including home visitors, parenting interventionists, and mental health therapists, address the quantity and quality of these caregiving experiences in their direct intervention with families. Additionally, IECMH providers can use such evidence in their consultation with the systems that serve these children, including health clinics, early care and education programs, community organizations, and, of course, child welfare agencies.

## 2 CONCLUSIONS

Including and retaining infants and young children in research that systematically examines the effects of TIC and early substance exposure and life stress, while also exploring protective factors and resilience processes, is essential for understanding how environments influence development. HBCD is a unique, longitudinal population‐based study that addresses these complex phenomena. Ultimately, such studies are important for science and society for theoretical and empirical reasons, especially for the field of infant and early childhood neurodevelopment and mental health. Meaningful inclusion and retention of children who experience TIC requires attention to state child welfare policies and practices, consent/permission issues, recruitment and retention challenges, diversity, equity, and inclusion, and methods that document transitions and flexibly follow children as they grow older. In so doing, the HBCD study can be used to inform future research that aims to capture children's early TIC and to identify factors that mitigate risk and promote resilience, which has important practice implications for the IECMH field.

## CONFLICT OF INTEREST STATEMENT

The Children's Hospital of Philadelphia has received payment for the expert testimony of Dr. Chaiyachati when subpoenaed for cases of suspected child maltreatment. Dr. Das reported that he is a shareholder of Lasso Informatics.

## APPRECIATION FOR DIVERSITY

The HEALthy Brain and Child Development (HBCD) study's research questions and methods reflect diversity in the sample. In addition, the paper includes a team of diverse scholars, including those who reflect the lived experiences of the sample to be studied. HBCD investigators stress the importance of inclusive and anti‐stigmatizing language, policies, and procedures, especially for families who experience substance use or involvement in the child welfare system.

## Data Availability

HBCD data are shared through curated public releases from the National Institutes of Health starting 2025 (https://hbcdstudy.org/data‐sharing/).

## APPENDIX B

### SOP for Engagement of Participants with Potential Child Welfare Involvement in HBCD

1. PAUSE

In studies involving pregnant persons and children, research staff should pause before presuming parenting behavior is maltreating (especially regarding neglect) and before making a report of child abuse or neglect.

a. Implement an internal mechanism to
i. Reflect on the facts and reasons for reporting.
ii. Reflect on what are the actual policies about reporting and the actual requirements (at the institution, the state, and the county).
iii. Reflect on stereotypes and biases that exist and lead to over‐reporting against some groups (families living in poverty, families of color).
iv. Reflect on the potential consequences of reporting and not reporting.
b. Ensure that research staff engage in discussion with a supervisor or colleague before, during, and after making a report.
v. Understand that child maltreatment reporting is emotionally challenging for the participant and for the staff.
vi. Be prepared for participant response to reporting (e.g., anger, leaving the study).
vii. Focus on the safety of reporters, as even anonymous reports may be sometimes blamed on research staff.
viii. Refer to statements of professional organizations regarding mandated reporting.
2. INCLUSION, SENSITIVITY, and SELF‐CARE
a. Understand the importance of words related to families impacted by child welfare, criminal legal systems, and substance use. Words can be highly stigmatizing (see relevant documents from the National Institutes of Health entitled “words matter”
i. https://nida.nih.gov/nidamed‐medical‐health‐professionals/health‐professions‐education/words‐matter‐language‐showing‐compassion‐care‐women‐infants‐families‐communities‐impacted‐substance‐use‐disorder
ii. https://nida.nih.gov/research‐topics/addiction‐science/words‐matter‐preferred‐language‐talking‐about‐addiction
b. Build in opportunities to provide support and facilitate self‐care for all staff
i. Breathing exercise or meditation as part of workshops or presentations
ii. Find a way to take breaks
iii. Acknowledge emotions of all staff
iv. Provide additional support for study navigators
c. Including people with lived experience/expertise in planning, implementation, decision‐making, and education is critical
i. Attend to how this is done (e.g., power hierarchies, authenticity, relationship‐building)
ii. Acknowledge potential triggers, both to people with lived experience/expertise and to wider audience
3. SUPPORT

Be mandated supporters first, then mandated reporters.

a. Treat all people in a supportive manner.
i. Evidence on child welfare involvement underscores the many contextual risks that involved families face.
1. Provide referrals for concrete and income supports.
2. Provide referrals for psychological and mental health resources.
3. Attempt warm hand‐offs when families are experiencing acute trauma.
  i. Evidence on secondary traumatic stress points to the emotional toll of engaging with families who have experienced trauma.
1. Regular reflective supervision for staff to address the emotional consequences of engaging maltreating families.
2. Consistent focus on staff safety and mental health.
  ii. Continue training and supervision regarding unconscious bias, stigma, and working with special populations (link to diversity, equity, and inclusion work).

### Child Welfare Expertise

1. KNOWLEDGE DEVELOPMENT

Staff at all levels (e.g., RAs, PIs, students) should be well‐informed about the child welfare system and ethical considerations in this area.

a. Provide on‐boarding and ongoing training about mandated reporting conducted by an institutional expert and child welfare system employee.
b. Be clear on state, institutional, and local policies re: reporting.
c. Reach out to HBCD Legal and Transitions in Care Workgroups for assistance.
d. Encourage staff to attend webinars and utilize resources on child welfare system involvement (see website of Administration of Children and Families: acf.hhs.gov).
e. Identify HBCD staff who has (or is willing to develop) child welfare expertise.
2. COLLABORATION WITH CHILD WELFARE SYSTEM:
a. Identify child welfare system liaison from every recruitment jurisdiction to facilitate study participation for child welfare involved families.
i. Ideal if liaison can serve on Community Advisory Board (CAB).
ii. Regular meetings with liaison are critical if they do not serve on CAB.
b. Include parent with lived experience of child welfare system on site CAB, even if it is on a second CAB that meets separately as a way to be sensitive to potential power dynamics.
c. Establish a Memorandum of Understanding with the child welfare system from each recruitment jurisdiction (e.g., how to handle reports, following children in foster care). In some places, however, this may not be feasible, depending on the child welfare system's capacity for involvement in outside research or the system's structure (e.g., different agencies for each county in a state).

### Research and Data Collection

1. DATA

Capture as much data as possible relevant to participants’ child welfare experience.

a. when a child abuse or neglect report is made by research staff and the outcome of the report, if known (often this is not known).
i. Document these occurrences in site‐specific logs.
b. when a child transitions from one caregiver to another, including formal foster care placement
i. Transitions questions are included in specific visits.
c. when children are in foster care
i. Complete brain and neurocognitive assessments
ii. Administer child functioning (e.g., social‐emotional functioning) and caregiving questionnaires/assessments to other caregivers (e.g., ERICA)
d. when participants are referred to prevention, “alternative response”, or other programs to address child maltreatment.
i. Document such referrals in study navigator referral form.
ii. Document such referrals in site‐specific logs.
2. RECORDS

Collection of child welfare system records

- Child maltreatment investigation outcomes (e.g., substantiation, foster care placement)
- Foster care placement (e.g., change in caregiver, services received)
- Ancillary studies should be conducted at sites that have strong relations with child welfare and have the willingness and ability to collect child welfare data.
